# Supplementary material for: Serial Lift-Out: sampling the molecular anatomy of whole organisms
Source: Nat Methods. 2023 Dec 18;21(9):1684–92. doi: 10.1038/s41592-023-02113-5 (PMC11399102; doi:10.1038/s41592-023-02113-5)
Supplement: Supplementary file 1 — Supplementary Figs. 1–3 and Note 1. [file 41592_2023_2113_MOESM1_ESM.pdf]

nature methods

Article

https://doi.org/10.1038/s41592-023-02113-5

Serial Lift-Out: sampling the molecular anatomy of whole organisms

In the format provided by the authors and unedited

Supplementary Information

Table of Contents

Supplementary Figure 1 2
Supplementary Figure 2 3
Supplementary Figure 3 4
Supplementary Movie 1 5
Supplementary Movie 2 5
Supplementary Movie 3 5
Supplementary Files 5
Step by Step Protocol 6

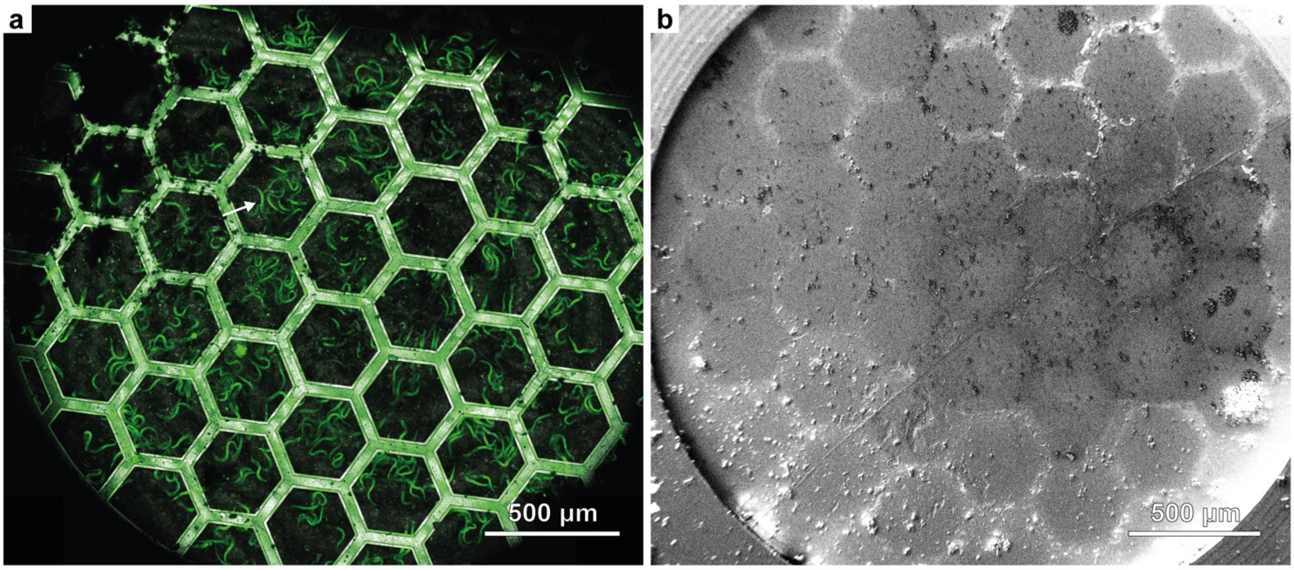

Supplementary Figure 1: Overview of the 'waffle'-type grid used for the double-sided attachment Serial Lift-Out. a, Maximum-intensity projection of the fluorescence (green) and reflected light (grey) channels **b**, SEM overview of the grid shown in (**a**).

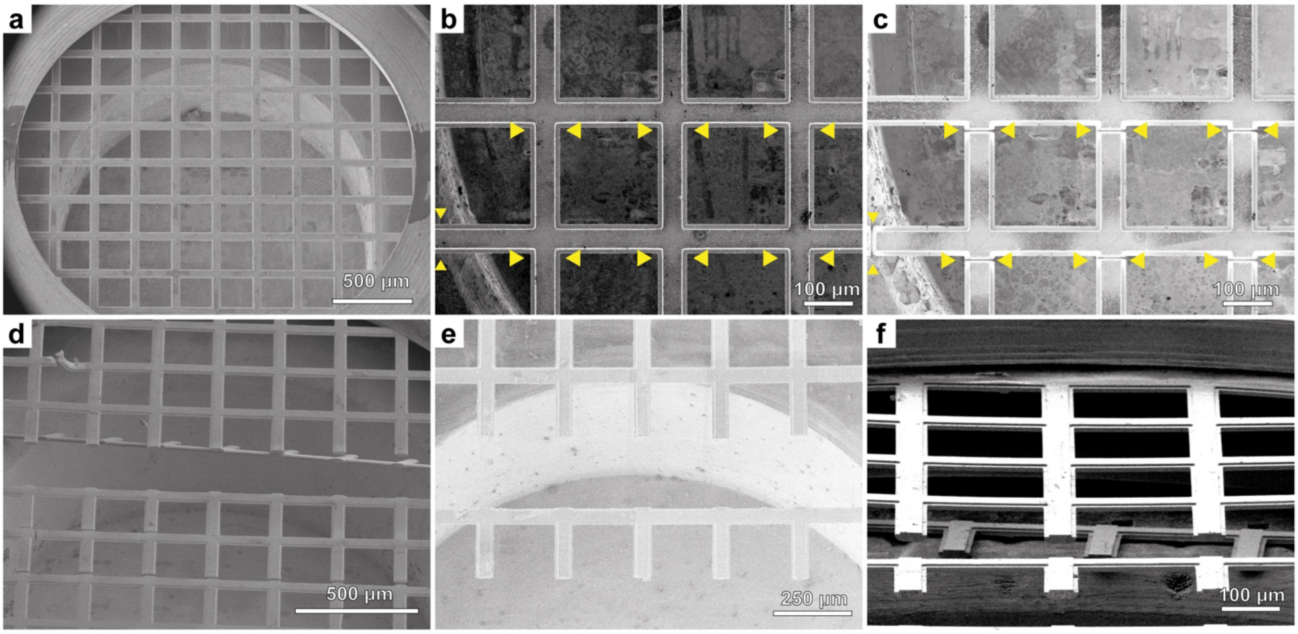

Supplementary Figure 2: Preparation of a Serial Lift-Out receiver grid for single-sided attachment. a, SEM image view of a 100 square mesh copper grid clipped into a cartridge. **b**,**c**, FIB images of grid bars prior to (**b**) and after (**c**) milling line patterns (between yellow arrowheads, trench milling orientation, beam current 65 nA). **d**, SEM image of the first grid bar after complete milling. **e**,**f**, SEM (**e**) and FIB (**f**) view of the final grid after both grid bars were removed (lamella milling orientation).

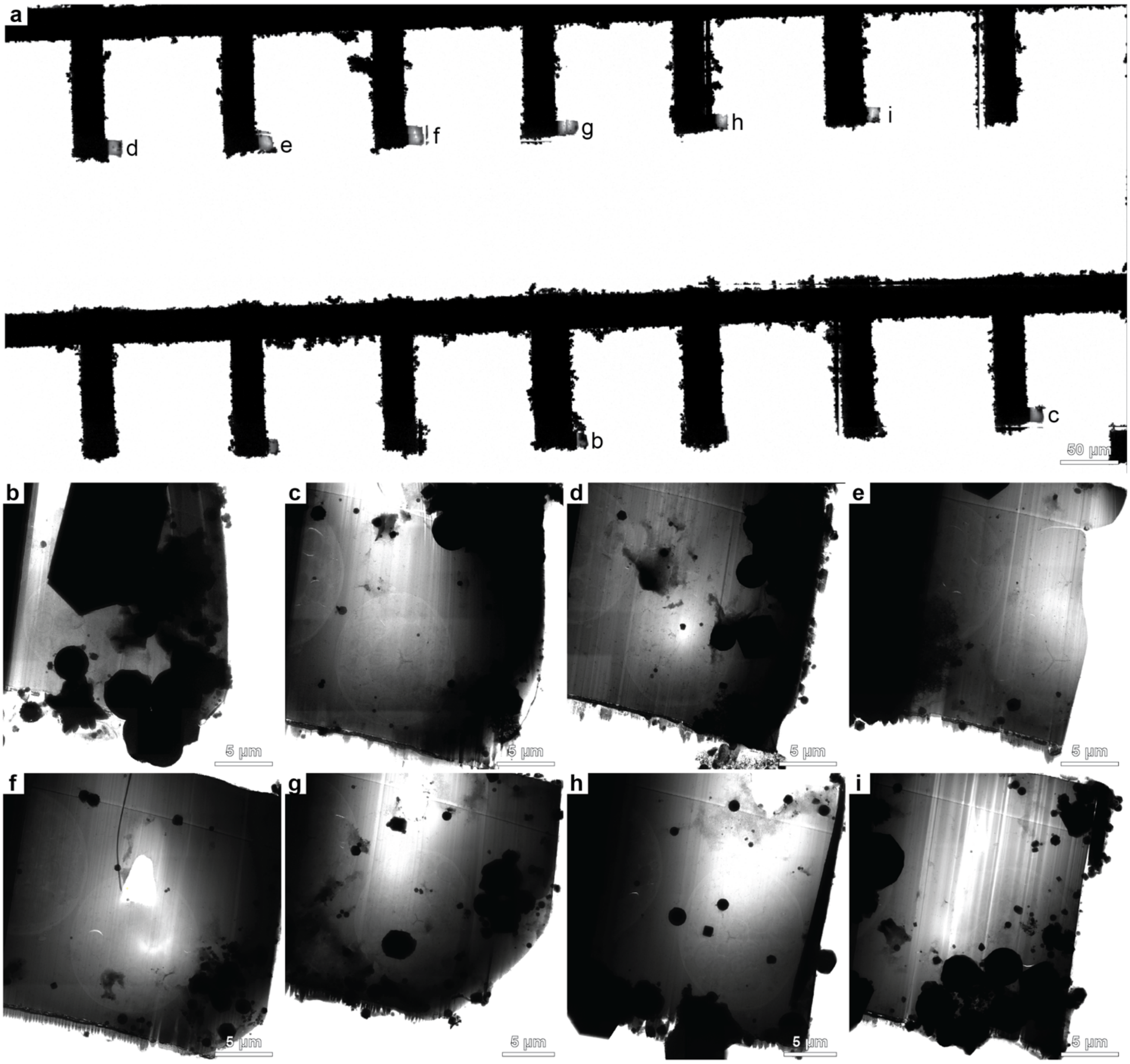

Supplementary Figure 3: TEM overview of the grid derived from single-sided attachment Serial Lift-Out. **a**, TEM low magnification overview image (magnification 125x) of the lamella region of the Serial Lift-Out receiver grid. **b-h**, Lamella montage overviews (magnification 11,500x) of 8 out of 12 fine-milled lamellae that survived transfer into the TEM and on which tilt series were acquired. The letters in panel **a** indicate the corresponding higher magnification micrographs in panels **b**-**h**.

Supplementary Movie 1: Double-sided Serial Lift-Out workflow. A movie summarizing the Serial Lift-Out process with double-sided attachment. The L1 larva was targeted by correlating a fluorescence overview to the grid surface. Lift-out is performed from the trench milling orientation. Copper redeposition is used to attach the adapter to the extraction volume. The volume is released by milling a line pattern and the EasyLift needle is retracted. The stage is set to lamella milling orientation on the receiver grid, and the needle is reinserted. The extracted volume's lower edge is aligned to a previously milled line mark (line pattern across the bars) and redeposition from the grid bars is used to attach the volume. The bottom section is released from the remaining extracted volume using line pattern milling. The process of attachment and sectioning was repeated in order to create 40 serial lift-out sections.

Supplementary Movie 2: Single-sided Serial Lift-Out workflow. A movie summarizing the Serial Lift-Out process with single-sided attachment. The L1 larva was targeted by correlating a fluorescence overview to the grid surface. Lift-out is performed from the trench milling orientation. Copper redeposition is used to attach the adapter to the extraction volume. The volume is released by milling a line pattern and the EasyLift needle is retracted. The stage is set to lamella milling orientation on the receiver grid, and the needle is reinserted. The extracted volume's lower edge is aligned to a corner of the pin and redeposition from the pin is used to attach the volume. The bottom section is released from the remaining extracted volume using line pattern milling. The process of attachment and sectioning was repeated in order to create 12 serial lift-out sections.

Supplementary Movie 3: Morphological detail on intermediate magnification overview maps. The movie illustrates the amount of detail, that can be extracted from lamella overview montages recorded at 11,500x magnification. Cross-sections obtained from the double-sided attachment Serial-Lift-Out experiment are shown. The first section stems from the procorpus, the anterior pharynx region (Extended Data Figure 6e). In the subsequent sections (Extended Data Figure 6f-j), the central pharynx widens up to form the anterior pharyngeal bulb or metacorpus. The seventh section in the movie was taken at the anterior pharyngeal isthmus and contains the anterior dorsal part of the nerve ring. Here, a zoomed in view of the nerve ring illustrates the discernible detail. Section 8 of the movie (Extended Data Figure 6l) is successive to the previous. The camera zooms in on the pharynx, the posterior ventral part of the nerve ring, a golgi apparatus, body wall muscle cells, a lateral amphid process bundle and a seam cell.

Supplementary Files: Thermo Fisher Scientific FIB-SEM instrument pattern files. The ZIP-Folder contains pattern files (.ptf) for various Serial Lift-Out steps: 1. Attachment of the copper block to the needle (Block_to_needle_glue.ptf), 2. Attachment of the copper block to the extraction volume (Block_to_bio_glue.ptf), 3. Double-sided attachment using a 100/400 rectangular mesh copper grid (Double_sided_attachment.ptf) and 4. Single-sided attachment using pins generated from a 100 square mesh copper grid (Single_sided_attachment.ptf). Schematics of the patterns are shown in Extended Data Figure 10.

Step by Step Protocol

Step-By-Step Towards Successful Serial Lift-Out

Authors

Oda Helene Schiøtz*[,1], Christoph J.O. Kaiser*[,1], Sven Klumpe*[,#,1], Dustin R. Morado[2], Matthias Poege[3], Jonathan Schneider[3], Florian Beck[1], David Klebl[2], Christopher Thompson[4], Jürgen M. Plitzko[#,1]

[1] Research Group CryoEM Technology, Max Planck Institute of Biochemistry, Martinsried, Germany
[2] Department Cell and Virus Structure, Max Planck Institute of Biochemistry, Martinsried, Germany
[3] Department Molecular Structural Biology, Max Planck Institute of Biochemistry, Martinsried, Germany
[4] Materials & Structural Analysis, Thermo Fisher Scientific, Eindhoven, The Netherlands

* Equal contribution
# Correspondence to: klumpe@biochem.mpg.de, plitzko@biochem.mpg.de

Keywords

C. elegans, L1 larva, Lift-Out, Cryo-ET, tomography, tissue, vitrification, high pressure freezing, lamella, preparation, ultrastructure, *in situ*, structural biology

Abstract

Cryo-focused ion beam milling of frozen-hydrated cells and subsequent cryo-electron tomography (cryo-ET) has enabled the structural elucidation of macromolecular complexes directly inside cells. The applicability of the technique to multicellular organisms and tissues is, however, still limited. While high-pressure freezing enables the vitrification of thicker samples, it prolongs subsequent preparation due to increased thinning times and the necessity to repeatedly perform complex and time-consuming procedures. Additionally, sample thinning removes large portions of the specimen, restricting the imageable volume to the thickness of the final lamella, typically < 300 nm. To resolve this, we developed a method termed 'Serial Lift-Out', an enhanced lift-out technique that increases throughput and obtainable contextual information by preparing multiple sections from single transfers. Here we describe in a step-by-step fashion the actions required to successfully perform such an experiment, deploying vitrified *C. elegans* L1 larvae from a 'waffle'-type sample, as an example to obtain a series of lamellae.

Introduction

Single particle analysis (SPA) by cryo-transmission electron microscopy (cryo-TEM) has become a key technique to study the structure of isolated biological macromolecules at high-resolution

(Kühlbrandt, 2014). This reductionist approach of studying protein complexes *in vitro*, however, loses all information concerning their molecular sociology: the interaction of the molecular complexes in their natural environment (Beck & Baumeister, 2016). Conversely, *in situ* cryo-electron tomography (cryo-ET) allows for the reconstruction of pleomorphic structures such as the crowded interior of the cell at molecular resolution, maintaining the interaction and localization of protein complexes within the biological system (Dietrich *et al.*, 2022, Gupta *et al.*, 2021, Hoffmann *et al.*, 2022, O'Reilly *et al.*, 2020, Plitzko *et al.*, 2017, Watanabe *et al.*, 2020).

One of the primary factors limiting the resolution of cryo-TEM is inelastic scattering. As the mean free path of an electron in vitrified biological samples is about 300-400 nm, samples beyond the size of viruses and small prokaryotic cells are generally too thick for cryo-ET (Liedtke *et al.*, 2022, O'Reilly *et al.*, 2020). Today, sample thinning in a focused ion beam (FIB) instrument at cryogenic temperatures has been widely adopted by the community. While not completely damage-free (Berger *et al.*, 2023, Lucas & Grigorieff, 2023), the technique has been shown to yield data that can allow for the elucidation of ribosomes sub-4Å resolution (Hoffmann *et al.*, 2022). The automation of lamella preparation by cryo-FIB milling has also reduced the need for user expertise and manual intervention (Buckley *et al.*, 2020, Klumpe *et al.*, 2021, Zachs *et al.*, 2020).

Prior to thinning, the sample must be cryogenically fixed by cooling at a sufficiently high rate to prevent ice crystal formation, resulting in a vitrified sample. There are two main methods available for vitrification: plunge freezing, in which the sample is immersed at ambient pressure into liquid ethane or ethane-propane mixture (Tivol *et al.*, 2008), and high pressure freezing (HPF), in which the sample is cooled with a jet of liquid nitrogen at a pressure of ~2000 bar. While the former yields samples that are easily FIB-milled, the sample thickness that can reliably be vitrified is limited and only the smallest cells vitrify entirely. HPF, on the other hand, allows for the vitrification of samples up to a thickness of roughly 200 µm (Dubochet, 1995)

Consequently, HPF greatly expands the size range of biological samples that can be vitrified but comes at the cost of embedding the specimen in a thick layer of ice defined by the depth of the freezing receptacle. This increased sample thickness leads to longer milling times. While samples up to a thickness of about 50 µm can be prepared by milling lamellae directly on the grid following the 'waffle' method (Kelley *et al.*, 2022) or alternative freezing approaches using 2-methyl pentane (Harapin *et al.*, 2015, Zhu *et al.*, 2022), lamellae from thicker samples have, to date, only been prepared by cryo-lift-out (Klumpe *et al.*, 2021, Kuba *et al.*, 2021, Mahamid *et al.*, 2015, Parmenter *et al.*, 2016, Rubino *et al.*, 2012, Schaffer *et al.*, 2019).

Cryo-lift-out refers to the extraction of the material for lamella preparation from bulk HPF sample and subsequent transfer and attachment to a lift-out receiver grid, conventionally a half-moon shaped grid (Giannuzzi *et al.*, 2005). Initially, trenches are milled around the area of interest, leaving it connected to the bulk material on a single side. For specimens in HPF sample carriers, the material must additionally be cleared from below. After these preparatory steps, the lift-out device, a micromanipulator-mounted tungsten needle, is attached to the volume to be extracted. The remaining connection to the bulk material is removed, the micromanipulator is used to transfer the extracted volume, and redeposition milling (Schreiber *et al.*, 2018) is used to attach the volume to the receiver grid. Finally, a lamella < 300 nm in thickness is prepared (Parmenter & Nizamudeen, 2021).

A limitation to cryo-lift-out, as well as on-grid lamella preparation, has been the loss of contextual information. Only a tiny fraction of the sample volume (< 1% for larger eukaryotic cells, << 1% for multicellular specimens) ends up inside the final lamella for cryo-ET data acquisition. In this protocol, we describe a novel cryo-lift-out approach that creates a series of lamellae from one lift-out volume that we term Serial Lift-Out. Inspired by diamond knife serial sectioning, Serial Lift-Out retains more contextual information than previous procedures and increases the throughput of cryo-lift-out by an order of magnitude.

Before embarking on using this protocol, the experimenter should be familiar with the usage of HPF equipment, cryo-fluorescent light microscopy, cryo-FIB/SEM and cryo-TEM, and should have established routines and strategies in place for tilt series acquisition and tomogram reconstruction. Although *C. elegans* is autofluorescent, which can be used to spot individuals to lift-out from a 'waffle' grid, it is recommended to choose a fluorescent marker strain, as it helps in localization and specific targeting of a tissue of interest. It is also a good idea to book/plan for additional time for the first Serial Lift-Out experiments and consider possible points of interrupting the workflow. Good points to interrupt the workflow are: after trenching, after lift-out and sectioning, and after fine-milling. We recommend to not interrupt milling after having achieved a lamella thickness ≤ 1 µm, as thinner sections have a tendency to deform and damage during sample transfers. In general, avoid unnecessary sample transfers, especially once the lamellae have been fine-milled. The experimenter should also consider that the lamellae contain a lot of – potentially interesting – biological features, so extended TEM time is needed to record the tilt series. If only e.g. 24 h timeslots are available, fine-milling only several sections, recording data on those, returning to the FIB-SEM and milling down the next batch of sections may be a viable solution. The drawback of this iterative milling approach is that the grid contaminates during the loading/unloading cycles, which might interfere with milling or data acquisition

Before embarking on a lengthy Serial Lift-Out session of a sample, it is advisable to mill a few 'waffle'-type lamellae or perform a lift-out session producing only few lamellae, that may be used to confirm the vitrification state of the sample, recording a handful of tilt series.

This protocol describes Serial Lift-Out on a single trenched extraction volume, targeted either by correlation only or aided by an integrated fluorescence microscope from 'waffle'-type L1 larva samples with double-sided attachment using a Thermo Fisher FIB-SEM system with an EasyLift micromanipulator and a Thermo Fisher Scientific cryo-stage.

Reagents and equipment

Equipment

Type	Details	Catalogue no.
Centrifuge for 1.5 mL caps	e.g. Eppendorf 5418 R	5401000010
Centrifuge for falcon tubes	e.g. Eppendorf 5910 Ri	5943000011
Grids, 100 mesh (single-sided attachment) or<br>Grids, 100/400 mesh (double-sided attachment)	e.g. Graticules	[1GC 100] 07D00903 or [1GC 100/400] 07D00917
Grids, low mesh number	e.g. Graticules copper 50 square mesh	[1GC 50] 07D00901
High pressure freezer	e.g. Leica Microsystems EM ICE	16771801
Petri dishes	e.g. Sarstedt petri dish, 60 x 15 mm, with ventilation cams, sterile	82.1194.500
Sample carriers type B, 6 mm, gold-plated copper	Leica Microsystems	16770182
Sample holder half cylinder for high pressure freezer	Leica Microsystems	16771847
Sample holder middle plates, 6 mm, for high pressure freezer	Leica Microsystems	16771834
Type 3 tweezers<br>(preferably isolated)	e.g. PELCO ESD Safe Tweezers	5083
Worm pick	Pt/Ir wire (Fisher Scientific 0.152 mm, hard), melted into a glass rod, flattened on the free end	11349498
Autogrid tweezers	Thermo Fisher Scientific	943290997631
C-Clip rings	Thermo Fisher Scientific	1036173
C-Clips	Thermo Fisher Scientific	1036171

Clipping base/station	Thermo Fisher Scientific	
Clipping tools	Thermo Fisher Scientific	
Cryo FIB/SEM microscope with rotatable stage and needle-based lift-out system	Thermo Fisher Scientific Aquilos or Aquilos2 with Easylift system	
Cryo grid boxes	Thermo Fisher Scientific	1084591
Cryo light microscope or integrated fluorescence microscope	Leica TCS SP8 or Delmic METEOR system	
Cryo transmission electron microscope	e.g. Thermo Fisher Scientific Krios G3	
Grid box opening tool	Thermo Fisher Scientific	
Large and intermediate size tweezers	For handling of grid boxes, sample carriers and sample holders in cryo and at RT	
Bunsen burner		
Falcon tubes 15 mL		
Incubator (20°C)		
Pasteur-type glass pipette		

Reagents

Reagent	Source	Catalogue no.
E. coli strains OP50 or HB101	Ceaenorhabditis Genetics Center at the University of Minnesota or Promega	N/A
Worm strain of interest	Ceaenorhabditis Genetics Center at the University of Minnesota	N/A
1,2-dichloroethane	Fluka, p.a.	03530
Bacto Agar	Beckton Dickinson	214010
Bleach (household bleach)	e.g. Dan Klorix, take a brand, that has no added agents except for bleach	N/A
$CaCl_2$	Roth, water free	A119.1
Cetyl palmitate	Roth, synthetic	9229.2
Cholesterol	Thermo Scientific, 95% stabilized	110190250
Ficoll 400	Sigma	F4375
Formvar	TCI, Polyvinylformal	P0641
KH_2PO_4	Roth, p.a.	3904.1
$MgSO_4.7H_2O$	Merck, p.a.	5886
Na_2HPO_4	Supelco, p.a.	1.06586
NaCl	Roth, p.a.	3957.2
NaOH	Merck, p.a.	1.06482
NH_4Cl	Supelco, p.a.	1.01145
Sucrose	Millipore, Microbiology grade	1.07651
Acetone	Technical grade	
Diethyl ether	Merck, p.a.	
Ethanol	Technical grade	
LB medium powder		
LN_2		

Solutions

Type	Details
10 M NaOH	In H_2O
1 M $CaCl_2$ (sterile)	In H_2O
1 M $MgSO_4$ (sterile)	In H_2O
1 M KH_2PO_4 (sterile)	In H_2O
5 mg/mL cholesterol	In ethanol, sterile filter with membrane that withstands organic solvent.
LB medium (sterile)	Dissolve in H_2O, aliquot in reagent tubes and flasks, autoclave.
Nematode growth medium agar, rich (sterile)	Dissolve 3g $NaCl_2$, 7.5g Bacto-Peptone, 21g Agar in H_2O and autoclave. When cooled to 55° C, add successively while stirring 1 mL cholesterol (5

	mg/mL), 1 mL 1M $CaCl_2$, 1 mL $MgSO_4$, 25 mL KH_2PO_4) pour into plates without introducing air bubbles.
M9 Medium (sterile)	Dissolve 5.8g Na_2HPO_4, 3g KH_2PO_4, 0.5g NaCl, 1g NH_4Cl, in water, aliquot to 200 mL and autoclave.
Sucrose solution, 60% (w/v, sterile)	Dissolve Sucrose in H_2O and autoclave.
Ficoll 400 solution 40% (w/v)	Dissolve Ficoll 400 in M9 medium. This usually requires vigorous shaking and centrifugation or prolonged incubation at elevated temperature.
Cetyl palmitate solution 0.5% (w/v)	Dissolve cetyl palmitate in diethyl ether.
Sample carrier cleaning solution	Mix equal volumes of household Bleach and 10 M NaOH solution. Prepare before use.
Bleaching solution	3 mL household Bleach, 0.4 mL 10 M NaOH, fill to 15 mL with H_2O. Prepare before use.
Ethanol 80% (v/v)	

Procedure

Here we provide a detailed protocol for the serial lift-out method, as described in the accompanying paper (Schiøtz *et al.* 2023). For stage orientation definitions and additional experimental details, please confer this publication.

Sample generation

1. Cultivate *E. coli* OP50 in LB medium. For a 2 L batch of NGM, prepare 250 mL bacteria solution by inoculating from a 5 mL starting culture. Seed NGM plates after 2 days of resting in a safety workbench and incubate at 37 °C overnight. The plates can be stored at 4 °C for several weeks.
2. Cultivate the worms according to standard procedures on 5 seeded 6-cm petri dishes with rich NGM (Stiernagle, 2006).
3. When the plates are densely populated but not starved yet, i.e. the plate mainly has gravid adult hermaphrodites, synchronize the worms by bleaching:
    a. Add M9 medium to plates and allow worms to dislodge from the agar for 15-20 mins. If plates soak up all liquid, add more M9 medium. Try to keep the total volume on all 5 plates below 15 mL.
    b. Swirl the plates and pipet the solution into a 15 mL falcon tube. Worms will start to settle in the tube. If there are still adults on the plate, take some of the liquid in the falcon tube and wash the plate by forcefully pipetting the liquid back onto the plate to remove it again. Repeat until most worms are collected.
    c. Centrifuge at 175x g for 1 min. If supernatant is clear, continue with step 2.d, if supernatant is still turbid, remove M9, fill with fresh M9, shake and spin down again. Repeat until supernatant remains clear. Supernatant is best removed by connecting a sterile Pasteur-type glass pipette to a suction flask connected to a vacuum line.
    d. Remove all supernatant without disturbing the worm pellet.
    e. Add bleaching solution.
    f. Keep rocking the tube and check regularly under a binocular too assess progress of the reaction. Continue until roughly half of the adults appear broken, but not for longer

than 6 min. Increase bleach concentration or use new bleach, if adequate bleaching would take more time.

g. Immediately centrifuge at 175x g for 1 min.
h. Immediately remove supernatant and fill with M9 medium.
i. Spin at 175 x g for 1 min.
j. Remove supernatant and fill with M9 medium.
k. Repeat steps i. and j. for at least 5 more times.
l. Leave 1 mL of solution in the tube after the last centrifugation step.
m. Put the supernatant on a rolling wheel or a shaker for 24 h at 20 °C to allow for hatching and synchronization.

4. Count larvae and add 700-800 L1 larvae to new NGM plates.
5. Cultivate at 20°C until gravid adults dominate the population (normally roughly 3 days at 20 °C).
6. Bleach as in step 3. Take utmost care not to overbleach the eggs. Washing with M9 can be reduced to 3 repeats as, after floating on sucrose, the eggs will be washed again. Leave 5 mL of M9 medium after the last bleaching wash.
7. Float on sucrose, adapted from Strange *et al.*, 2007:
    a. Resuspend the egg pellet in the remaining 5 mL of M9 medium.
    b. Add an equal volume (5 mL) of 60% (w/v) sucrose solution (sterile) to the egg solution and mix well.
    c. Centrifuge for 4 min at 350x g.
    d. Remove from centrifuge without disturbing the layering.
    e. Using a plastic Pasteur pipette or a standard 1 mL displacement pipette, remove the uppermost layer of liquid, containing the eggs (~3 mL). If eggs stick to tube walls, wash them off with some of the sucrose solution in the tube.
    f. Add an excess amount of M9 medium and mix.
    g. Centrifuge for 4 min at 350x g.
    h. Remove supernatant. Repeat steps f-h twice.
8. Remove all liquid to yield < 1mL of egg solution.
9. Put the supernatant on a rolling wheel or a shaker for 24 h at 20 °C to allow for hatching and synchronization.

Vitrification

1. Coat the high-pressure freezing sample carriers (Type B high-pressure freezing sample carriers) with cetyl palmitate. This is should be done in a fume hood.
    a. Clean the sample carriers if previously used with sample carrier cleaning solution. Any other harsh cleaning that removes hydrophobic organic compounds and hydrophilic

compounds will work on gold-plated sample carriers. Leave the sample carriers in cleaning solution overnight followed by three water and three acetone washing steps.

   b. Dry the sample carriers.
   c. Dip the sample carriers, one at time, into the cetyl palmitate solution, quickly shaking off excess liquid.
   d. Place the carriers on filter paper, cavity side down, to let the ether evaporate and allow the hydrophobic coating to form. There should be a slight haze on the sample carrier, only clearly visible under the binocular. If the layer is clearly visible with the naked eye, the layer is likely too thick.

2. Prepare formvar-coated grids. These grids are also available from commercial suppliers but can be prepared on-site. A number of protocols are available online. The thickness of the film is of minor importance when freezing 'waffle'-type samples.
   a. Dissolve 1% (w/v) formvar in 1,2-dicloroethane in a bottle.
   b. Dip an object slide previously cleaned with window cleanser into the solution.
   c. Remove the slide from the solution and allow excess formvar solution to drain for 10 s while leaving the slide in the solvent vapor phase.
   d. Air-dry the slide.
   e. Score the film on both sides with a razor blade roughly 1 mm from the top, the bottom and the sides.
   f. Dip the slide slowly into water in a dark container. The film should float off from both sides. The process can be supported pulling slightly with tweezers. If the film doesn't float off, change the cleaning procedure, experimenting with different agents.
   g. Place grids (shiny side down) onto the film.
   h. Recover the coated grids by placing a piece of parafilm on top of them, and push everything below the water surface at an angle. When the whole formvar layer is submerged, pull the parafilm out and air dry in a petri dish.
3. Prepare 40% (w/V) Ficoll solution in buffer or medium (here in M9). We usually prepare 1-1.5 mL in a 1.5 mL tube.
   a. Weigh the appropriate amount of Ficoll 400.
   b. Add roughly 50%-70% of the final volume of solvent (e.g. sterile M9 medium).
   c. Shake vigorously. Ficoll hydrates slowly. If the solution is prepared ahead of time, incubate while shaking overnight in a thermoblock at 42 °C. If the solution is immediately needed, centrifuge briefly, resuspend stirring with a 10 µL pipet tip and centrifuge again. Repeat until all Ficoll is dissolved.
   d. Adjust to target volume.

4. Dilute an aliquot of the 40% Ficoll solution to 20 % with the medium of choice (e.g. M9 medium). 3 µL of this solution are needed per frozen grid, so 50 µL of this solution should be sufficient.
5. Cool down the high-pressure freezer. Prepare HPF sample holder half cylinders, middle plates and tools.
6. Cool down the sample dewar.
7. Spin down the synchronized L1 larvae (200x g for 2 min). Remove supernatant to obtain less than 200 µL of animal suspension. Transfer into PCR tube and spin again. Remove supernatant to obtain a dense suspension of animals.
8. Add an equal volume of 40% Ficoll to the worm ssuspension and mix by gently pipetting (200 µL tip).
9. Place the sample holder half cylinders and a middle plate in the appropriate cavities of the Leica EM ICE.
10. Place a piece of filter paper in front of the lower sample holder.
11. Place a coated Type B sample carrier with its cavity down on the filter paper.
12. Add a 3 µL drop of 20% Ficoll to the middle of the coated sample carrier.
13. Place a formvar-coated grid, film side down, onto the drop. Make sure all the formvar is in contact with the Ficoll solution.
14. Blot away the drop using a filter paper placed below the edge of the grid. Try to remove as much liquid as possible without introducing air below the grid, leaving a minimal liquid film, ensuring the formvar lies flat on the carrier.
15. Add 3 µL of worms in 20 % Ficoll onto the grid.
16. Use tweezers or the pipette tip to distribute worm solution to all grid squares of the grid.
17. Use tweezers to remove as many air bubbles as possible. Dip the tweezers into the liquid and poke the bubbles to make them float to the surface. Alternatively, opening the tweezers will produce a liquid column between its tips, into which bubbles can float and be lifted from there lifting the tweezers.
18. Add the second sample carrier with its cavity side up onto the worm drop.
19. Press using the tweezers and wick off expelled sample droplets.
20. Move the sandwich to the sample holder middle plate, keeping the sandwich aligned.
21. Press on the sandwich with the back side of the tweezers for 10 s and immediately close the lid of the sample chamber.
22. Repeat steps 9-21 until all samples are frozen.
23. Cool down the sample handling station.
24. Remove the sample dewar from the EM ICE and release the lower part into the sample handling station.

25. Remove all plastic parts, just leaving sample carriers and middle plates still containing sample carriers. Disinfect all the parts by directly placing them into 80% ethanol. Soak all plastic parts before repeated use in H_2O overnight to remove Ficoll contaminations.
26. Release the carriers from middle plates by pushing them out. If necessary, split sample carrier sandwiches.
27. Collect all sample carriers with a grid attached and all grids that became detached. Remove all other middle plates and empty sample carriers from the handling station. Transfer detached grids to a grid box for storage.
28. To release grids, with the grid side facing up, push down on the sample carrier at 3, 6, 9 and 12 o'clock positions on the carrier close to the grid rim with closed type 3 tweezers. This breaks the ice layer and should release the grid. If the grid does not release, push the sample carrier against a ridge and push the grid rim with closed tweezers at roughly 30-45° elevation angle. Do not press only laterally but with equal downwards force.
29. Place the grids into grid boxes for storage in LN_2.

Note: Insufficiently clean sample carriers or too thick coating with cetyl palmitate will interfere with grid release. A good checkpoint for coating quality is when the Ficoll solution below the grid is placed on the sample carrier. When this drop spreads out at low contact angle, it crawls below the cetyl palmitate layer, indicating the coating quality is insufficient. Try cleaning more rigorously by adding a washing step (e.g. 10 % SDS and/or heating the cleaning solution). Re-coat the sample carriers. Bad coating often originates from a too old and/or too concentrated cetyl palmitate solution. In this case, a new coating solution should be prepared. A properly coated sample carrier will hold the Ficoll drop at high contact angle.

Fluorescence data acquisition

1. Start up and cool down the cryo fluorescence light microscope (e.g. Leica SP8).
2. Clip grids. 'Waffle' grids may need more force to clip than plunge-frozen grids.
    a. Fill the loading or clipping station with dry LN_2.
    b. Transfer a grid box containing the 'waffle' samples from storage to the station.
    c. Mark a C-clip ring with a red permanent marker line (6 o'clock) and two black permanent marker lines (3 and 9 o'clock) and load it into the recess of either the clipping base or clipping station. The markings will aid orientation during subsequent loading steps.
    d. Place a 'waffle' grid in the groove of the C-clip ring.
    e. Load a clipping tool with a C-clip and cool thoroughly before using the tool to clip the C-clip into the C-clip ring.
    f. Repeat steps c to e until all grids which are to be screened during the session are clipped.

3. Load the samples into the cryo-confocal microscope. If the samples were not clipped in a clipping base within the Leica SP8 loading station,
    a. Cool the loading station with dry LN_2 and transfer a grid box with grids to this station.
    b. Use a pair of pre-cooled AutoGrid tweezers to load the grids into the Leica SP8 shuttle in such a way that the red marking is located around 6 o'clock. The Leica SP8 is otherwise loaded as stated in the manufacturer's manual.
4. Use the oculars to determine the rough focus position.
5. Set excitation and emission wavelengths.
    a. Set excitation laser and emission wavelength for the fluorophore of interest.
    b. Set excitation laser and emission wavelength to the same value in order to detect reflection. If there is an emission filter such as a Leica Fluorifier in place, test, whether it has to be set to 'empty' to obtain reflection images.
    c. Set up a transmission channel.
    d. Adjust laser intensity and detector gain on a typical sample area for all channels such that the resulting signal is neither over- nor underexposed. Be careful not to oversaturate sensitive detectors such as avalanche photodiodes or hybrid detectors.
6. Acquire an initial single-slice tiled overview map of the whole grid at low optical zoom. Decrease scan speed if necessary and open up the pinhole completely.
7. Set optics to the acquisition settings: close pinhole to desired value, increase scan speed and optical zoom to achieve the level of detail needed for the project. Switch on bidirectional scanning and adjust phase.
8. Set a number of evenly spaced focus points (~15-20) across the grid. Focus on the grid bars in the reflection channel and set the focus for each point.
9. Localize an animal preferably close to a grid bar using the fluorescent channel.
10. Determine the z-stack dimension by focusing through the sample in reflection and fluorescence. Start the stack slightly beyond the reflection of the far air/ice interface and the end beyond the near air/ice interface reflection to include the whole sample volume in the z-dimension of the stack. For an average waffle grid (e.g. 50 mesh) the start and end planes should be spaced roughly ~30-50 µm. Set the reflection of the grid bar as focus plane in the stack.
11. Re-map the grid by recording z-stacks in a tiled fashion. Increase optical slice spacing in the z-direction to keep mapping times below 2 h.
    a. If more precise targeting is needed, record site-specific z-stacks using higher resolution imaging parameters.
12. Montage the tiled overview and calculate maximum projections for all channels. These maps provide clear signal that can be used for 2D correlation, with the sample surface in the FIB-SEM instrument.

13. Unload the sample and store the grids in LN_2 for further use.
14. Repeat steps 3-13 for all grids that are to be screened.

Preparatory steps

This section summarizes steps that need to be performed on the FIB-SEM to prepare for the Serial Lift-Out experiment such as needle preparation, copper block attachment and receiver grid preparation. These steps can also be executed at room temperature. Stage tilt angles assume the use of a 45° pretilt FIB shuttle and a 52° angle between SEM and FIB column.

1. Prepare the lift-out needle. Before beginning a lift-out session, check the state of the lift-out system (e.g. the Easylift). This is best done at room temperature to allow lift-out needle exchange if necessary.
    a. Focus the electron beam on a position on the shuttle and link the stage.
    b. Choose a position on the shuttle and use it to adjust the z-height so that the position is at the beam coincidence point.
    c. Move the shuttle down by 2-3 mm.
    d. Insert the needle and examine its state. If the needle is bent, exchange the needle as described in the user manual.
    e. For this method the needle should be milled so that it has a flat lower face about 10-20 µm wide. If this is not already the case, mill away the needle tip using a cleaning cross-section at 30 kV and 15 nA.
    f. If the system has not been used recently or the needle has been just been exchanged, check the needle movement range in z. If the range is too small, execute the needle calibration procedure (within the alignments tab). Note: if this calibration is done at room temperature, calibrate the system such that the needle tip extends 25 µm diagonally past the center of the screen as that is the expected shrinkage of the needle during cooling. If calibration is without success, exchange or readjust the needle in the holder. It is also good to double check the calibration once the system has been cooled.
    g. Select the lowest magnification in the FIB channel and move the needle tip in z to just below the upper image edge.
    h. Retract the needle.
2. Clip a receiver grid.
    a. Using a clipping station or clipping base at room temperature, load a C-clip ring.
    b. Place a 100/400 mesh (double-sided attachment) or 100 mesh (single-sided attachment) Cu grid in the C-clip ring groove in such a way that the shiny side faces down and the dull side faces up.
    c. Load a clipping tool with a C-clip and clip the grid into place.

d. Use a red and black permanent marker to mark the AutoGrid. For the 100/400 mesh grid, a red marking is placed in line with the 400-mesh grid bars and two black markings are placed in line with the 100-mesh grid bars. For the 100-mesh grid, the markings should be in line with the grid bar directions. These aid in future orientation steps.

3. Load the receiver grid into the FIB/SEM.
   a. For the 100/400 mesh grid, align the mesh such that the 400-mesh bars run vertically within the shuttle the 100-mesh bars run horizontally. Use a magnifying glass, if hard to see. For the 100-mesh grids, align the grid such that the grid bars run vertically and horizontally in the shuttle. In both cases the red marking should be at 6 o'clock and the black lines 3 and 9 o'clock.
   b. Load the microscope as otherwise described in the manual.
4. Prepare a copper block for attachment to the needle. FIB currents up to 65 nA can be used on gallium FIB systems.
   a. Mill down a copper bar of the receiver grid in lamella milling orientation (0° relative rotation to loading angle, 18° stage tilt) to obtain a grid bar area that is roughly 20 µm thick and 70-80 µm wide. Use 15 to 65 nA beam current and regular cross-sections to speed up the process.
   b. Rotate the grid to trenching orientation (180° relative rotation to loading angle, 7° stage tilt).
   c. Mill the previously milled section of the grid bar to generate a chain of blocks that are roughly 10 µm x 20 µm in size. Leave them connected on their sides to form a chain of two to three blocks, but well separated from the bulk copper (cf. Schiøtz *et al.* 2023, Figure 1 – Figure Supplement 2). Generating a trench of roughly 3-5 µm is essential, as otherwise redeposited material in the release step (step h) may re-attach the copper to the grid bar.
   d. Return the stage to lamella milling orientation (0° relative rotation to loading angle, 18° stage tilt) and adjust the z-height so that the blocks are at the coincidence point.
   e. Insert the needle and maneuver it on top of one of the blocks. Using the electron beam, adjust the needle so that its attachment face protrudes from the copper block. This ensures proper redeposition for attachment.
   f. Bring the needle in close contact with the block's top face. If the surfaces do not properly align, polish the faces using cleaning cross-sections to make sure both surfaces are properly flat.
   g. Attach the block to the needle through redeposition milling on the copper block directed away from the attachment site (x-array of regular cross-sections, single pass, width 0.5 µm, lateral spacing 0.25 µm, height 2.5 µm, z-depth 4 µm, 30 kV, 300 pA). Place the milling start of the patterns on the interface of the copper block and the needle. Note

the importance of the cross-section patterns being set to single pass. Multipass set to more than 1 will ablate material that has been redeposited.

h. Release the block by milling the remaining attachment using regular cross-sections (30 kV, 15 nA). Move the needle initially only in a step-wise fashion (e.g. 200 nm) to check for release. Mill longer and closer to the copper block, if it has not been released.
i. Once released, select the lowest magnification in the FIB channel and use z to move the copper block to just below the upper image edge.
j. Retract the needle

5. Prepare the reciever grid:
   a. Double-sided attachment reciever grid:
      i. With the 100/400 mesh Cu grid loaded as stated in Step 3, rotate the stage to trench milling orientation (180° relative rotation to loading angle, 7° stage tilt).
      ii. Mill a horizontal line pattern (e.g. 30 kV, 15 nA, z-depth 5 µm) across the 100-mesh grid bars, such that the line sections them at around 1/3 from the top of the grid. This should be done for the middle 3-4 rows of the grid and extend over 15 grid meshes. The lines should mark the grid, but not cut through the metal.
   b. Single-sided attachment grid:
      i. With the 100-mesh Cu grid loaded as stated in Step 3, rotate the stage to trench milling orientation (180° relative rotation to loading angle, 7° stage tilt).
      ii. Create pins by milling out every other grid bar. Line patterns are milled (30 kV, 50 nA, z-depth 20 µm) to be able to remove every other grid bar, generating e.g. two rows of 7 pins or three rows of 5 pins.

Trench Milling

Trench milling is described as it was performed on an Aquilos 2 system and on an Aquilos 1 system, equipped with a METEOR integrated light microscope. This protocol should, however, be adaptable to most cryo-FIB-SEM microscopes. The dimensions for the extraction volume are chosen to allow for double-sided attachment using 100/400 mesh grids. Stage tilt angles assume the use of a 45° pretilt FIB shuttle and a SEM to FIB column angle of 52°.

Two variants exist for this task: **Variant A** exclusively deploys the previously recorded fluorescence/reflection grid map for targeting. **Variant B** initially uses the very same map for rough targeting, but also deploys the integrated light microscope to render targeting more precise. Note: while variant B can be performed without prior fluorescence screening, we still recommend recording a grid map, as screening large areas of the sample with an integrated light microscope systems is time-consuming.

1. Start up and cool the FIB-SEM instrument.
2. Cool down the loading station with dry LN_2.

3. Transfer the pre-screened grids to the loading station and load them into the shuttle.
    a. Load so that the red marking is located at 6 o'clock to match the orientation of the light microscope map and the orientation in the FIB-SEM instrument as closely as possible.
    b. If, after trench milling, the lift-out and slicing procedure is supposed to happen in the same session, additionally load a receiver grid as described in the section 'preparatory steps', step 3.
4. Load the shuttle into the FIB-SEM microscope.
5. Focus on the sample and link the working distance.
6. Set the stage to lamella milling orientation (0° relative rotation to loading angle, 18° stage tilt). If there is excessive contamination, scan with the ion beam (30 kV, 100 pA) while rapidly increasing and decreasing magnification to remove contaminant ice particles.
7. Set the stage to the imaging orientation (0° relative rotation to loading angle, 45° stage tilt) and record a low magnification high resolution electron image for mapping (e.g., 5kV, 13 pA, 6144x4096 pixels, dwell time 2 µs).
8. Set the stage to trench milling orientation (180° relative rotation to loading angle and 7° stage tilt, ion beam normal to the sample surface). Lower the stage to a stage working distance of 10.6 mm and apply a layer of protective organometallic platinum using the gas injection system heated to 27 °C. An exposure of 90 s is split into three cycles of 30 s each with a 15 s pause between cycles. The exposure time can vary due to the distance of the gas injection system needle to the sample surface affecting the deposition rate. If required, adjust the time to obtain a platinum layer of 1-2 µm.
9. Set the stage to the imaging orientation (0° relative rotation to loading angle, 45° stage tilt) and acquire a low magnification high resolution SEM overview image (e.g., 5kV, 13 pA, 6144x4096 pixels, dwell time 2 µs).
10. Correlate the overview images and the previously acquired fluorescence/reflection maps using either MAPS (Thermo Fisher Scientific), 3DCT (https://3dct.semper.space/) or other image analysis software. Grid bars and other surface features that can be localized in the reflected light channel, the initial overview and the overview obtained after platinum coating are used to correlate the images.

    For variant A: This step is crucial in the absence of an integrated fluorescence microscope and decisive for faithful targeting.
    For variant B: The correlation can be locally imperfect, because the integrated fluorescence microscope will be used to precisely locate the region of interest.
11. Use the correlation to select regions of interest, e.g. relatively straight *C. elegans* L1 larvae.
12. Set the stage to the trench milling orientation (180° relative rotation to loading angle, 7° stage tilt, ion beam normal to the sample surface).

For variant A: Acquire a low magnification FIB image (5 kV, 10 pA, 3072x2048 pixels, dwell time 500 ns) and correlate it with care to the last low magnification SEM image and the previously acquired fluorescence data.

13. Adjust the coincidence point at the region(s) of interest.
14. **Variant A:** Determine the position of the region of interest precisely, using surface features visible in the correlated FIB image. Do this with great care, as this step determines the targeting precision.

    Variant B:
    a. Mill cross-shaped markers (two intersecting line patterns e.g. 2 µm long, z-depth 1 µm, 30 kV, 100 pA or a regular cross-section or rectangular pattern with similar parameters) near the region of interest. The markers should not be within the region of interest, so keep sufficient distance.
    b. Acquire a lower magnification FIB image of these regions (5 kV, 10 pA, 3072x2048 pixels, dwell time 500 ns). The field of view should contain the entire region of interest and the milled markers in order to do proper correlation with the subsequently recorded fluorescence data.
    c. Move the sample to the fluorescence data acquisition position.
    d. Acquire overview tile sets and/or z-stacks of the region(s) of interest in fluorescence and reflection modes if available. Note: in order to acquire reflection data with the METEOR system one of the emission filters needs to be removed or left empty during installation.
    e. Correlate the recorded light microscopy data to the previously recorded FIB image using the milled markers and/or surface features visible in the reflected light or fluorescence channel.
    f. Determine the region of interest precisely, using the markers and surface features.
15. Mill three trenches (30 kV, 3 nA) around the region of interest.
    a. Mill an upper trench about 50-60 µm wide and 30-40 µm high in order to allow for sufficient room to maneuver the needle to the extraction volume. This trench should be shifted off-center from the region of interest in the direction of the incoming needle.
    b. Mill the two side trenches at the length of the region of interest and 3-5 µm in width. Note: one trench can be a line pattern, reducing milling time, however, wider trenches facilitate the extraction process and make it easier to judge whether the whole depth of the 'waffle' was fully ablated.

       For variant B: Move the sample to the fluorescence data acquisition position and acquire images for the region of interest to confirm proper targeting and re-adjust trenches moving back to the FIB position.
    c. Polish the extraction volume's top face at a lower current (e.g. 300 pA) to remove the high beam current damage and smoothen the face for attachment. The final

dimensions of the block should be the length of the region of interest by the width of the grid meshes +2 µm (e.g. 40-42 µm).

16. Set the stage to the imaging orientation (0° relative rotation to loading angle, 45° stage tilt) and acquire a low magnification high resolution SEM overview image (e.g., 5kV, 13 pA, 6144x4096 pixels, dwell time 2 µs). This image is used for orienting the grid when re-loading for lift-out, in case the session is interrupted.
17. If not performing lift-out and sectioning immediately, unload the shuttle into a pre-cooled loading station and store the grid in LN_2.

Lift-Out

Steps 1-6 only need to be performed if the sample was stored after trench milling. Otherwise proceed from step 7. Stage tilt angles assume the use of a 45° pretilt FIB shuttle. Ensure, the needle was prepared for this step (cf. section ‘preparatory steps’, step 1 and 4)

1. Cool down the FIB-SEM instrument.
2. Cool down the loading station with dry LN_2.
3. Transfer the sample and the receiver grid into the shuttle.
    a. Use the SEM image taken of the grid after trench milling in order to orient the grid such that the trench is aligned upright within the shuttle. Note: if the trenches were milled at an angle, this will mean that the red marking is no longer located at 6 o’clock.
    b. Load the receiver grid as described in the section ‘preparatory steps’, step 3.
4. Load the shuttle into the FIB-SEM microscope.
5. Focus on the sample to link the stage.
6. Set the stage to trench milling orientation (180° relative rotation to loading angle, 7° stage tilt).
7. Set the coincidence point at the region of interest.
8. If the grid was stored after trenching, try to mill away potential contaminants.
9. Insert the needle into the chamber.
10. Using the FIB and SEM channels to monitor movement, maneuver the needle into the top trench to bring the copper block in contact with the top face of the lift-out volume. Ensure that the copper block’s front face is aligned behind the front face of the extraction volume (i.e. roughly in the middle of the volume’s top face) to allow for proper redeposition attachment.
11. Attach the copper block to the extraction volume through redeposition milling on the copper block directed away from the attachment site (horizontal array of regular cross-sections, single pass, width 0.5 µm, lateral spacing 0.25 µm, height 2.5 µm, z-depth 4 µm, 30 kV, 300 pA). Place the top end of the patterns on the interface of the copper block and extraction volume. Note the importance of the cross-section patterns being set to single pass. If Multipass is set to more than 1, the ion beam will scan the redeposition region multiple times and will consequently ablate material that has been redeposited.

12. Mill a line pattern across the lower part of the extraction volume to release it from the bulk material (30 kV, 1 nA, z-depth 10 µm).
13. Move the needle in a stepwise fashion (200 nm) once in z and y to check if the extraction volume was properly released.
14. Repeat steps 12 and 13 until the extraction volume is clearly detached from the bulk sample.
15. Lift the extraction volume out of the plane of the 'waffle'-grid. Monitor the extraction process with both SEM and FIB images. Start by moving in z, then move y. Movement out of the grid plane in y may have a z component, so iteratively move z and y and compensate x if necessary.
16. Once well above the grid plane, select the lowest magnification in the FIB channel and use z to move the extracted volume to just below the upper image edge.
17. Retract the needle.

Sample transfer and sectioning

Stage tilt angles assume the use of a 45° pretilt FIB shuttle.

1. Move the stage to position the receiver grid in the field of view.
2. Set the stage to lamella milling orientation (0° relative rotation to loading angle, 18° stage tilt) and adjust the stage rotation to make sure that the pins or 400 mesh grid bars are aligned vertical.
3. Set up the coincidence points for all positions to be used for section attachment. Place them in the middle of the field of view and save the positions. If corrections of rotation are necessary to have perfectly vertically running 400-mesh grid bars or pins, perform them during this step and save them with the stage positions. Note: assuming the receiver grid is perfectly loaded, saving a single coincidence point per row may suffice.
4. Go back to the first section attachment position.
5. Re-insert the needle to which the extracted volume is attached.
6. Lower the needle so that the extracted volume is about 10 µm above the first position.
7. Position the extracted volume using the SEM channel.
    a. For double-sided attachment, adjust y to align the leading edge of the volume with the previously milled line pattern and adjust x to place the volume precisely between the two grid bars.
    b. For single-sided attachment, adjust y to align the lower front edge of the volume with the front edge of the pin and adjust x to align the left or right side of the block with the side of the pin. Note: both sides of the pins can be used for single sided attachment by alternating the side of attachment of the extracted volume.

8. Lower the extraction volume into place by adjusting z. Use the FIB channel as guidance. Double-check intermittently for alignment with line/corner landmarks using the SEM channel.
    a. For double sided attachment:
        i. If the extraction volume is too wide to fit between the grid bars, mill off excess material using regular cross sections or patterns (30kV, 300 pA). The extracted volume should fit close to perfectly into the mesh.
        ii. Align the lower front edge of the extraction volume with the previously milled line.
    b. For single sided attachment, align the lower front edge of the extraction volume with the nearest corner of the pin.
9. Attach the extracted volume to the grid bars by redeposition milling. Place the milling start of the patterns on the interface of the grid bar and extraction volume.
    a. For double-sided attachment mill on both adjacent grid bars for attachment. Mill a vertical array of regular cross-sections (single pass, width 4.0 µm, height 0.5 µm, z-depth 10 µm, vertical spacing 0.25 µm, 30 kV, 1 nA) directed away from the extracted volume.
    b. For single-sided attachment mill on the pin for attachment. Mill a vertical array of regular cross-sections (single pass, width 5.0 µm, height 0.5 µm, z-depth 5 µm, vertical spacing 0.25 µm, 30 kV, 1 nA) directed away from the extracted volume.
10. Move the needle up by a step of 50-100 nm to create strain.
    a. For single sided attachment also move 50 nm in y away from the grid bar.
11. Release the section by milling a line pattern across the extracted volume at the desired sectioning distance from the lower edge of the extracted volume (30kV, 1 nA, z-depth 20 µm). Sectioning at 4 µm is recommended to begin with, but sections down to 1 µm can be obtained.
12. Check whether the section has been released by moving the needle up by 1-3 steps of 50 nm. If the section moves with the needle, repeat the line pattern milling.
13. Once the section is released, carefully maneuver the extraction volume up. As soon as there is some distance to the section (~1 µm), increase the step size or jog. Move the volume up slightly below the edge of the FIB image in lowest magnification
14. Retract the needle.
15. Move the stage to the next section attachment position and continue from step 5. Repeat this, until the extracted volume has been completely sectioned.

After having prepared the sections, the grid can be stored indefinitely in LN_2.The sections can also be examined by fluorescence microscopy in order to accurately determine regions of interest within the section, reducing the time spent on section thinning.

Section thinning

Sections can be thinned to lamellae in a single or multiple sessions. The strategy depends on the time available for tilt series acquisition (cf. section 'time taken'). For L1 larvae, we typically thin 20-25 µm wide regions, which is sufficiently wide to accommodate the larval cross-section. While wider regions can be thinned, this increases the required milling time and leads to a higher probability of lamella deformation and consequently uneven thinning or lamella loss in the final thinning steps. Stage tilt angles assume the use of a 45° pretilt FIB shuttle.

If sections were not stored and are thinned to lamellae right after sectioning, start at step 7.

1. Cool down the FIB-SEM instrument.
2. Cool down the loading station with dry LN_2.
3. Transfer the sample into the shuttle as described in the section 'preparatory steps', step 3 for the receiver grid.
4. Load the shuttle into the FIB-SEM microscope.
5. Focus the sample and link the stage.
6. Set the stage to lamella milling orientation (0° relative rotation to loading angle, 18° stage tilt).
7. Set the coincidence point for all sections that are to be thinned during the session and save these stage positions.
8. Move the stage to the first section to be thinned.
9. Mill down to a thickness of 1.5 µm using regular cross-section patterns at 30 kV, 1 nA.
10. Mill down to a thickness of 1.2 µm using regular cross-section patterns at 30 kV, 0.5 nA.
11. Mill down to a thickness of 0.8 µm using regular cross-section patterns at 30 kV, 0.3 nA.
12. Move to the next section.
13. Repeat steps 9-12 until all desired sections have been thinned to 0.8 µm thickness.
14. Go back to the first section.
15. Mill down to a thickness of 0.4 µm using regular patterns at 0.1 nA.
16. Over- and under-tilt the stage by 1° and use regular rectangle patterns at 50 pA to even out the lamella thickness by milling the back of the lamella.
17. Mill down to a thickness of ~0.2 µm using regular patterns at 50 pA.
18. Move to the next lamella to be thinned.
19. Repeat steps 15-18 iteratively until all desired lamellae have reached their final thickness.
20. Sputter coat the lamellae with platinum for 4 seconds at a chamber pressure of 0.20 mbar and a current of 15.0 mA. Pt-particles that are generated in this step can be used for image alignment, tilt angle refinement and reduce sample charging.

After fine-milling the lamellae, we recommend to transfer the grid directly into a cryo-TEM to minimize contamination. Make sure that all LN_2 used for the transfer is dry and, if possible, perform the transfer in a humidity-controlled room.

Note: Automated routines (Buckley *et al.*, 2020, Klumpe *et al.*, 2021, Zachs *et al.*, 2020) can be deployed for the section thinning process.

Tilt series acquisition

Tilt series are acquired following previously described strategies (Hagen *et al.*, 2017).

Time

The time taken for the preparation of worm samples may vary, but should range between 1-2 weeks. Recovering worms from frozen stocks or starved plates ranges between 3 days to 1.5 weeks, dependent on how many individuals were present at the beginning. After the first bleaching, a synchronous population is produced that follows the canonical worm developmental cycle times. Consequently, after another 3-4 days, depending on the temperature, a population of gravid young adults are present. Roughly 20-40 hours after the second bleaching, high pressure freezing should be performed.

**Step**	**Time**
High pressure freezing	~2-3 h for 6-8 grids
Cryo-confocal fluorescence screening	1.5-2 h/grid
Preparatory steps	2-4 h
Trench milling	~2 h/region of interest
Lift-out	1-2 h
Sectioning	15-30 min/section
Section thinning	25 min/section

Steps such as high pressure freezing, cryo-confocal fluorescence screening and preparatory steps are fully independent from the rest of the workflow and need to be done only once for a grid or several Serial Lift-Out sessions. Trench milling can be performed in one session and the very same grid can be re-used for several Serial Lift-Out sessions, reducing the amount of time required for the actual Lift-Out workflow. Dependent on the reliability of the cooling of the FIB-SEM instrument and the desired degree of automation for section thinning, running the microscope overnight can greatly speed up the process. Typically, with these factors in mind, 30-40 sections will require roughly 5 working days, from high pressure freezing until the fine-milled lamellae.

TEM tilt series acquisition time greatly depends on whether the data collection is targeted to specific regions or whether the entire lamella area is of interest (i.e. as many tomograms as can be recorded). Using e.g. the Tomo 5 software (Thermo Fisher Scientific), the acquisition of tiled overview/search maps, setting up targeted positions and the acquisition of tilt series for 5 lamellae will require probably 30-40h. If more lamellae (> 30) were milled, setup and acquisition times for overview/search maps may already take 12 h. On average, we were able to collect in the order of 70 tilt series/day, corresponding to 4-5 lamellae/day with multi shot acquisition in Tomo5. Deploying higher throughput data acquisition procedures (Eisenstein *et al.*, 2023) is likely to accelerate data collection.

Anticipated results

This protocol should yield 10-40 lamellae suitable for high-resolution tilt series acquisition, which allow the reconstruction of tomograms and subsequent averaging of extracted subtomograms to yield *in situ* protein complex reconstructions. These reconstructions can reach resolutions in the nanometer regime and below (e.g., ribosomes, microtubules).

Acknowledgments

We thank Jonathan Wagner, Adriana Prajica, Philipp S. Erdmann, Johann Brenner, Meijing Li, Alexander Rigort, Bernhard Hampoelz, Martin Beck, and Reinhard Fässler for invaluable input and support. We thank the Caenorhabditis Genetics Center and Bloomington Drosophila Stock Center for providing *C. elegans* and *D. melanogaster* strains, respectively. This study used the infrastructure of the Department of Cell and Virus Structure at the MPI of Biochemistry. SK was supported by the International Max Planck Research School for Molecular and Cellular Life Sciences. JMP acknowledges funding from the Max-Planck Society.

Associated publication

Serial Lift-Out – Sampling the Molecular Anatomy of Whole Organisms. OHS Schiøtz, CJO Kaiser, S Klumpe, DR Morado, M Poege, J Schneider, F Beck, DP Klebl, C Thompson, JM Plitzko, Nature Methods

Protocol References

Beck, M. & Baumeister, W. Cryo-Electron Tomography: Can it Reveal the Molecular Sociology of Cells in Atomic Detail? Trends Cell Biol. 26, 825-837 (2016).

Berger, C.*, et al.* Plasma FIB milling for the determination of structures in situ. Nat Commun. 14, 629 (2023).

Buckley, G.*, et al.* Automated cryo-lamella preparation for high-throughput in-situ structural biology. J. Struct. Biol. 210, 107488 (2020).

Dietrich, H. M.*, et al.* Membrane-anchored HDCR nanowires drive hydrogen-powered CO fixation. Nature. 607, 823-830 (2022).

Dubochet, J. High-pressure freezing for cryoelectron microscopy. Trends Cell Biol. 5, 366-368 (1995).

Eisenstein, F.*, et al.* Parallel cryo electron tomography on in situ lamellae. Nat Methods. 20, 131-138 (2023).

Giannuzzi, L. A.*, et al.* FIB Lift-Out Specimen Preparation Techniques. In: Introduction to Focused Ion Beams: Instrumentation, Theory, Techniques and Practice. (eds. Giannuzzi, L. A. & Stevie, F. A.) 201-228 (Springer US, Boston, MA, 2005).

Gupta, T. K.*, et al.* Structural basis for VIPP1 oligomerization and maintenance of thylakoid membrane integrity. Cell. 184, 3643-3659.e3623 (2021).

Hagen, W. J. H., *et al.* Implementation of a cryo-electron tomography tilt-scheme optimized for high resolution subtomogram averaging. J Struct Biol. 197, 191-198 (2017).

Harapin, J., *et al.* Structural analysis of multicellular organisms with cryo-electron tomography. Nature Methods. 12, 634-636 (2015).

Hoffmann, P. C., *et al.* Structures of the eukaryotic ribosome and its translational states in situ. Nat Commun. 13, 7435 (2022).

Kelley, K., *et al.* Waffle Method: A general and flexible approach for improving throughput in FIB-milling. Nat. Commun. 13, 1857 (2022).

Klumpe, S., *et al.* A modular platform for automated cryo-FIB workflows. Elife. 10, (2021).

Kuba, J., *et al.* Advanced cryo-tomography workflow developments - correlative microscopy, milling automation and cryo-lift-out. J. Microsc. 281, 112-124 (2021).

Kühlbrandt, W. Biochemistry. The resolution revolution. Science. 343, 1443-1444 (2014).

Liedtke, J., *et al.* How advances in cryo-electron tomography have contributed to our current view of bacterial cell biology. J Struct Biol X. 6, 100065 (2022).

Lucas, B. A. & Grigorieff, N. Quantification of gallium cryo-FIB milling damage in biological lamella. bioRxiv. 2023.2002.2001.526705 (2023).

Mahamid, J., *et al.* A focused ion beam milling and lift-out approach for site-specific preparation of frozen-hydrated lamellas from multicellular organisms. J. Struct. Biol. 192, 262-269 (2015).

O'Reilly, F. J., *et al.* In-cell architecture of an actively transcribing-translating expressome. Science. 369, 554-557 (2020).

Parmenter, C. D. & Nizamudeen, Z. A. Cryo-FIB-lift-out: practically impossible to practical reality. J. Microsc. 281, 157-174 (2021).

Parmenter, C. D. J., *et al.* Making the practically impossible "Merely difficult"--Cryogenic FIB lift-out for "Damage free" soft matter imaging. Microsc. Res. Tech. 79, 298-303 (2016).

Plitzko, J. M., *et al.* Structural Biology outside the box-inside the cell. Curr. Opin. Struct. Biol. 46, 110-121 (2017).

Rubino, S., *et al.* A site-specific focused-ion-beam lift-out method for cryo Transmission Electron Microscopy. J. Struct. Biol. 180, 572-576 (2012).

Schaffer, M., *et al.* A cryo-FIB lift-out technique enables molecular-resolution cryo-ET within native Caenorhabditis elegans tissue. Nat. Methods. 16, 757-762 (2019).

Schreiber, D. K., *et al.* A method for site-specific and cryogenic specimen fabrication of liquid/solid interfaces for atom probe tomography. Ultramicroscopy. 194, 89-99 (2018).

Stiernagle, T. Maintenance of C. elegans. WormBook. 1-11 (2006).

Strange, K., *et al.* Primary culture of Caenorhabditis elegans developing embryo cells for electrophysiological, cell biological and molecular studies. Nat Protoc. 2, 1003-1012 (2007).

Tivol, W. F., *et al.* An improved cryogen for plunge freezing. Microsc Microanal. 14, 375-379 (2008).

Watanabe, R.*, et al.* The In Situ Structure of Parkinson's Disease-Linked LRRK2. Cell. 182, 1508-1518.e1516 (2020).

Zachs, T.*, et al.* Fully automated, sequential focused ion beam milling for cryo-electron tomography. Elife. 9, (2020).

Zhu, H.*, et al.* In situ structure of intestinal apical surface reveals nanobristles on microvilli. Proceedings of the National Academy of Sciences. 119, e2122249119 (2022).
