# Supplementary material for: Serial Lift-Out: sampling the molecular anatomy of whole organisms
Source: Nat Methods. 2023 Dec 18;21(9):1684–92. doi: 10.1038/s41592-023-02113-5 (PMC11399102; doi:10.1038/s41592-023-02113-5)
Supplement: Supplementary file 2 — Reporting Summary [file 41592_2023_2113_MOESM2_ESM.pdf]

nature portfolio

Corresponding author(s): Sven Klumpe and Juergen M. Plitzko

Last updated by author(s): Sep 28, 2023

Reporting Summary

Nature Portfolio wishes to improve the reproducibility of the work that we publish. This form provides structure for consistency and transparency in reporting. For further information on Nature Portfolio policies, see our Editorial Policies and the Editorial Policy Checklist.

Statistics

For all statistical analyses, confirm that the following items are present in the figure legend, table legend, main text, or Methods section.

n/a	Confirmed	
☐	☒	The exact sample size (*n*) for each experimental group/condition, given as a discrete number and unit of measurement
☐	☒	A statement on whether measurements were taken from distinct samples or whether the same sample was measured repeatedly
☒	☐	The statistical test(s) used AND whether they are one- or two-sided<br>*Only common tests should be described solely by name; describe more complex techniques in the Methods section.*
☒	☐	A description of all covariates tested
☒	☐	A description of any assumptions or corrections, such as tests of normality and adjustment for multiple comparisons
☐	☒	A full description of the statistical parameters including central tendency (e.g. means) or other basic estimates (e.g. regression coefficient) AND variation (e.g. standard deviation) or associated estimates of uncertainty (e.g. confidence intervals)
☒	☐	For null hypothesis testing, the test statistic (e.g. *F*, *t*, *r*) with confidence intervals, effect sizes, degrees of freedom and *P* value noted<br>*Give P values as exact values whenever suitable.*
☒	☐	For Bayesian analysis, information on the choice of priors and Markov chain Monte Carlo settings
☒	☐	For hierarchical and complex designs, identification of the appropriate level for tests and full reporting of outcomes
☒	☐	Estimates of effect sizes (e.g. Cohen's *d*, Pearson's *r*), indicating how they were calculated

Our web collection on statistics for biologists contains articles on many of the points above.

Software and code

Policy information about availability of computer code

Data collection	FIB/SEM data: XT v 20.1.1<br>Confocal cryo fluorescence data: LAS X 3.5.5.19976<br>Wide field cryo fluorescence data: Odemis 3.2.1<br>Tilt series acquisition: Tomo5 5.12.0
Data analysis	Overview processing: ImageJ 2.9.0 + TrackEM plugin<br>Fluorescence data: MAPS 3.14<br>Tilt series/tomograms: IMOD 4.12.32, AreTomo 1.3.3, STOPGAP 0.7, TOMOMAN 0.7, Relion 4.0, Relion 3.0, Relion 3.1, WARP/M 1.0.9 , ChimeraX 1.3. MotionCor2 1.4.7, CTFFIND4 4.14

For manuscripts utilizing custom algorithms or software that are central to the research but not yet described in published literature, software must be made available to editors and reviewers. We strongly encourage code deposition in a community repository (e.g. GitHub). See the Nature Portfolio guidelines for submitting code & software for further information.

Data

Policy information about availability of data

All manuscripts must include a data availability statement. This statement should provide the following information, where applicable:

- Accession codes, unique identifiers, or web links for publicly available datasets
- A description of any restrictions on data availability
- For clinical datasets or third party data, please ensure that the statement adheres to our policy

Tomograms have been deposited in the Electron Microscopy Data Bank (EMDB) under accession codes: EMD-17246, EMD-17247, EMD-17248, EMD-18186 Subtomogram averages have been uploaded under accession numbers: EMD-17241, EMD-17242, EMD-17243, EMD-17244, EMD-17245, EMD-18187, and will be released upon publication.

Human research participants

Policy information about studies involving human research participants and Sex and Gender in Research.

Reporting on sex and gender	N/A
Population characteristics	N/A
Recruitment	N/A
Ethics oversight	N/A

Note that full information on the approval of the study protocol must also be provided in the manuscript.

Field-specific reporting

Please select the one below that is the best fit for your research. If you are not sure, read the appropriate sections before making your selection.

☒ Life sciences ☐ Behavioural & social sciences ☐ Ecological, evolutionary & environmental sciences

For a reference copy of the document with all sections, see nature.com/documents/nr-reporting-summary-flat.pdf

Life sciences study design

All studies must disclose on these points even when the disclosure is negative.

Sample size	Sample sizes were not determined through statistical methods. For the three separate C. elegans Serial Lift-Out and single on-grid experiments, four individuals from separate strains (twice AM140, both from one 'waffle' grid, twice NK2476 from one 'waffle' grid) were used. One egg chamber of Drosophila melanogaster (GFP-Nup358 endogenously tagged) was used for the Serial Lift-Out experiment from high-pressure freezing planchettes. Descriptive statistics on tomogram thicknesses is based on either all tomograms (n=56 for Experiment 1, n=90 for Experiment 3) or a randomized subset (n=132 for Experiment 2) of reconstructed tomograms for experiment .
Data exclusions	Tomograms for subtomogram averaging but not for tomogram thickness measurement were excluded based on on poor reconstruction quality and excessive thickness. Particles were excluded based on classification during subtomogram averaging when not contributing to an improvement of the GSFSC criterion.
Replication	Serial Lift-Out has been replicated several tens of times on different sample types by the authors and other members of the Baumeister and Briggs departments at the MPI of Biochemistry (MPI of Biochemistry, Martinsried, Germany). The method was adopted in other labs including the labs of Stefan Raunser (MPI for Molecular Physiology, Dortmund, Germany), Philipp Erdmann (Human Technopole, Milan, Italy), and Julia Mahamid (EMBL Heidelberg, Germany).
Randomization	Individuals of C. elegans were selected solely based on the criterion of accessibility for lift-out, mainly influenced by body curvature, lateral position and orientation on the grid. Tomograms for suntomogram averageing were only selected based on lamella thickness and otherwise randomly from the whole dataset.
Blinding	Data collection and analysis were not blinded.

Reporting for specific materials, systems and methods

We require information from authors about some types of materials, experimental systems and methods used in many studies. Here, indicate whether each material, system or method listed is relevant to your study. If you are not sure if a list item applies to your research, read the appropriate section before selecting a response.

Materials & experimental systems

n/a	Involved in the study
☒	☐ Antibodies
☒	☐ Eukaryotic cell lines
☒	☐ Palaeontology and archaeology
☐	☒ Animals and other organisms
☒	☐ Clinical data
☒	☐ Dual use research of concern

Methods

n/a	Involved in the study
☒	☐ ChIP-seq
☒	☐ Flow cytometry
☒	☐ MRI-based neuroimaging

Animals and other research organisms

Policy information about studies involving animals; ARRIVE guidelines recommended for reporting animal research, and Sex and Gender in Research

Laboratory animals	Caenorhabditis elegans, Drosophila melanogaster
Wild animals	No wild animals were used.
Reporting on sex	Hermaphrodites (C. elegans), female (D. melanogaster)
Field-collected samples	No field samples were used.
Ethics oversight	C. elegans and D. melanogaster do not require ethic approval for use in experiments.

Note that full information on the approval of the study protocol must also be provided in the manuscript.
